# The Body That Speaks: Recombining Bodies and Speech Sources in Unscripted Face-to-Face Communication

**DOI:** 10.3389/fpsyg.2016.01300

**Published:** 2016-09-08

**Authors:** Alex Gillespie, Kevin Corti

**Affiliations:** Department of Social Psychology, London School of Economics and Political ScienceLondon, UK

**Keywords:** communication, body, cyranoid, echoborg, speech shadowing, android science, experimental methods, avatar

## Abstract

This article examines advances in research methods that enable experimental substitution of the speaking body in unscripted face-to-face communication. A taxonomy of six hybrid social agents is presented by combining three types of bodies (mechanical, virtual, and human) with either an artificial or human speech source. Our contribution is to introduce and explore the significance of two particular hybrids: (1) the cyranoid method that enables humans to converse face-to-face through the medium of another person's body, and (2) the echoborg method that enables artificial intelligence to converse face-to-face through the medium of a human body. These two methods are distinct in being able to parse the unique influence of the human body when combined with various speech sources. We also introduce a new framework for conceptualizing the body's role in communication, distinguishing three levels: self's perspective on the body, other's perspective on the body, and self's perspective of other's perspective on the body. Within each level the cyranoid and echoborg methodologies make important research questions tractable. By conceptualizing and synthesizing these methods, we outline a novel paradigm of research on the role of the body in unscripted face-to-face communication.

## Introduction

Before paper, wires, and silicon, the primordial communication medium is the body. At the center of all communication rests the body, the fleshy gateway to the mind (Biocca, 1997, p. 13)

In face-to-face interaction, words seem inseparable from the bodies that speak them. Consequently, bodies fundamentally shape the intersubjectivity of social interaction. In human-human interaction, the age, gender, height, attractiveness, and other physical attributes of the speaking body mediate the meaning of utterances. For instance, if a young child were to suggest to you how to pull a country out of an economic recession, how would you react to their proposal compared to the same suggestions uttered by a scholarly-looking adult? Likewise, with regards to human-agent interaction, totally different qualities of intersubjectivity can be elicited by the same artificial intelligence depending on its embodiment. Imagine your different responses to the same chat bot if you encountered it embodied in a virtual avatar or speaking through a flesh and blood human body. Our program of research shows how people's interactions with the cognitive content of what is said is profoundly shaped by **the body that speaks**.

KEY CONCEPT 1 **The body that speaks**

Bodies are any animate medium that can be seen as the agent of communication. The most obvious bodies are human bodies, which can vary in terms of age, gender, height, size, attractiveness, and other visible attributes. Bodies, however, can also be human-like androids, robots, or representations in virtual reality, all of which can vary on a broader range of visible attributes.

In this article we demonstrate that six ostensibly disparate research methodologies, from human-computer interaction, social psychology, and communication, are fundamentally similar because they all experimentally separate and recombine **speech sources** and bodies in unscripted communication encounters. These methods are distinct because they primarily focus on animate bodies (as opposed to static or more minimal mediums, such as pictures and text) and real-time behavior (as opposed to questionnaire-responses to imagined social scenarios). Our own contribution has been to advance the cyranoid and echoborg methodologies (Milgram, 1992; Corti and Gillespie, 2015a), both of which are unique in their focus on examining the significance of the fleshy *human* body in communication. We then propose three conceptual levels for parsing the body's role in face-to-face conversation and at each level map-out paradigm of research questions that are made tractable by the cyranoid and echoborg methods.

KEY CONCEPT 2 **Speech source**

Speech sources are able to engage in conversation autonomously. Speech sources can utilize either human or artificial intelligence. Sources vary in terms of capability, expertise, understanding, emotional intelligence, and so on. Artificial speech sources include chat bots and even branching scripts, provided that they are elaborate enough to produce responses within real-time conversation.

## Mixing and merging bodies and speech sources

The taken-for-granted assumption that bodies author their own words is often unwarranted. First, people spend a lot of time reporting, directly and indirectly, the speech and beliefs of other people and groups (Bakhtin, 1986; Lucy, 1993; Aveling et al., 2015). This reported speech can borrow tone, facial expressions, and non-verbal gestures (Coulmas, 1986). Yet, the reported speech is also always new, being in a novel context, filled with distinctive motivation, and translated through a different body. The point is that much of what is said cannot be attributed to a solitary individual, rather, it entails hybrid authorship (Wertsch, 1991; Gillespie and Cornish, 2014). Moreover, there are situations in which the audience is not meant to know, or at least meant to forget, that the body that speaks is separate from the speech source: puppets are given voice by the puppeteer, actors speak words originating from the playwright, television anchors follow their teleprompter, and politicians ventriloquize the words and phrases authored by speechwriters. These widespread “mash ups” of bodies and speech sources, however, are mono-directional; reported speech, theater scripts, teleprompts, and speeches cannot adapt to real-time two-way conversation.

A more dynamic mixing and merging of bodies and cognition sources can be found in traditional beliefs. According to Hinduism, deities are able to manifest on earth, often as animals, called *avatars*. In Japanese folklore, foxes called *kitsune*, were thought to be wise and powerful beings capable of taking the form of men and women. Across Europe in the Sixteenth century, many people believed in werewolves, that is, people who could shape-shift into dangerous wolves. These beliefs and myths are powerful, troubling, and fascinating precisely because they question whether the body that speaks corresponds to the mind that understands.

Mixing and merging bodies and cognition sources has been a generative theme in theater, novels, and films. In Rostand's (1891/1981) 19th century play *Cyrano de Bergerac*, the protagonist woos Roxane by speaking to her through the more handsome body of Christian. In the novel *The Wonderful Wizard of Oz* (Baum, 1900/2000), the seemingly powerful wizard turns out to be a frail man behind a curtain with a voice amplifier who had gained authority by speaking through an imposing and seemingly powerful body. More recently, science fiction films have further explored the potentials of recombining bodies and minds. The films *Surrogates* (Banks et al., 2009) and *Avatar* (Kalogridis et al., 2009), for instance, focus on identity, status, and power transformation made possible by surrogate bodies, while films such as *Being John Malkovich* (Kaufman et al., 1999) have explored the implications of inhabiting another person's actual body. Another common theme, inspired by books such as *Do Androids Dream of Electric Sheep?* (Dick, 1968), has been whether a seemingly authentic human body that speaks is in fact guided by artificial intelligence. In each case, transgressing the audience's expectation that cognition belongs solely to the visible body opens up interesting plot lines and raises philosophical conundrums. Inspired by these themes, we ask: what research methodologies are available for recombining bodies and speech sources? And, what research questions can such methods address?

## Methodologies for recombining bodies and speech sources

We conceptualize three types of body, namely mechanical, virtual, and human. Each of these bodies can be paired with two basic types of speech source, namely, human or artificial. Each of the six resultant combinations corresponds to a distinctive methodological paradigm (outlined in Table 1).

**Table 1:** **Taxonomy of body and speech source combinations with associated methods**.

**Speaking body**	**Speech source**	**Methodological set-up**
Mechanical	Human	Tele-operated robot or android
Mechanical	Artificial	Autonomous robot or android
Virtual	Human	Avatar
Virtual	Artificial	Intelligent virtual agent
Human	Human	Cyranoid
Human	Artificial	Echoborg

### Tele-operated robots and androids

The first recombination entails pairing a human speech source with a mechanical body. Arguably, the earliest variants of this recombination were puppets (Jurkowski, 1996), notable amongst them being the original mechanical Turk (Standage, 2002). Technological advancements have enabled controlling ever more remote devices, such as submarines, drones, and even vehicles on other planets. However, it is only fairly recently that this capacity for long-distance tele-operation has been combined with two-way communication. For instance, the new field of android science uses tele-operated androids to merge human cognition sources with android bodies (MacDorman and Ishiguro, 2006). Devices known as “Geminoids” (tele-operated androids modeling specific humans) have been explored as doppelganger bodies for remote communicators. These devices involve a human operator controlling their android's speech and motor behavior via a remote console that recreates the android's audio-visual field. In some cases, tele-operation can be covert in nature such that the research participant is under the false impression that they are engaging with an artificial cognition source, and researchers will often intentionally prime this false assumption (known as the Wizard of Oz technique; Kelley, 1983). Though in many cases the Wizard of Oz technique is used in prototyping in order to circumvent limitations in artificial intelligence, it has also been used as a mechanism to evoke changes in social interaction.

The social psychological phenomena investigated through the use of tele-operated devices include concepts such as “presence” (the subjective sensation of being *with* another person; Nishio et al., 2007a) and the “uncanny valley” (Mori, 1970/2012). The uncanny valley hypothesis asserts that while people's affinity toward nonhuman physical bodies generally increases the more humanlike the body is, positive emotional evaluation will sharply decline if the body appears *too* human, yet still artificial. The concept has spurred speculation into its evolutionary origins (see Wang et al., 2015), but beyond being psychologically fascinating, the phenomenon has implications for how the integration of tele-operated devices in human society will unfold. Developers of devices intended to function as surrogate bodies in social interaction will want to optimize the physical attributes of their products so as to achieve the desired psychological response from users. It is likely that surrogate mechanical bodies will be used for office visits (Paulos and Canny, 1997), remote meetings (Tsui et al., 2011), and care visits (Tsai et al., 2007). As these technologies become more powerful, they will enable friends and family to not only visit and communicate with care-receivers, but also to engage in physical actions such as retrieving objects, cleaning, or making a meal. The uncanny valley phenomenon underscores the challenge of building a body that is acceptable to someone in need of care who is in a significant psychological relationship with that body (Michaud et al., 2007). What is clear from this research is that the body that speaks matters profoundly. For example, should the body be a Geminoid to facilitate identification, or, as Paulos and Canny (1997) suggest, should the body be deliberately artificial to avoid any confusion or mistaken attributions?

### Autonomous robots and androids

Swapping out the human tele-operator of a machine body for an artificial cognition source gives us the next species of hybrid: the fully *autonomous* android or robot. Though these agents are not hybrid in the sense of pairing separate artificial and human entities, they can be conceptualized as such in that they involve modifiable software (the cognition/speech source) paired to modifiable hardware (the body), thus allowing researchers to examine the role of embodiment on social interaction.

Software designed to enable a machine body to socially interact with a human range from low-level, language mimicry technology (e.g., chat bots) to high-level forms of artificial intelligence (Schumaker et al., 2007). Despite some technologies exhibiting remarkable domain-specific intelligence (e.g., IBM's Watson), a general artificial intelligence capable of allowing a machine body to fluidly replicate the full spectrum of human cognitive and motor capabilities remains elusive. As such, fully autonomous androids and robots are severely socially constrained in unscripted contextual and dynamic interactions with humans (Nishio et al., 2007b).

A machine-bodied autonomous entity fully identical to an organic human in all observable respects with regard to behavior, appearance, and communicative functioning, would pass what Harnad (2000) termed the “*Total* Turing Test” (see Turing, 1950). The path toward total human mimicry, however, will involve a series of incremental advancements, with each milestone bringing with it complex social psychological consequences. For instance, it remains to be seen whether and how humans will integrate advanced autonomous entities both psychologically and socially. These entities may eventually, in time, have an intelligence that far surpasses human intelligence (Bostrom, 2014), thus becoming psychologically incomprehensible to humans. These genuinely intelligent devices will likely replace humans in many occupations, creating the possibility of public resistance (Bauer, 2014). In such a future context, how these intelligent agents are embodied may be crucial for public acceptability.

### Avatars

Hybrids composed of virtual bodies (i.e., digital representations of bodies encountered on computer screens or within virtual reality environments) combined with human speech sources are known as avatars and have become increasingly useful in psychological research aimed at studying how the body transforms social perception and interaction (Blascovich et al., 2002). Avatars can range in sophistication (e.g., some are static representations of bodies while others exhibit complex motor behavior) and the extent to which they mimic human appearance (e.g., ranging from representing a specific human body to a fantastical non-humanoid body). Avatars are not only used for research; they are ubiquitous in digital media, providing playgrounds for identity (Evans, 2011).

One of the more sophisticated and productive methodologies for exploring social psychological phenomena by-way-of avatars involves the use of immersive virtual environment technology. This technology enables research participants to experience pre-constructed social situations in 3D via an avatar, and affords a significant degree of mundane realism (Blascovich et al., 2002). Within an immersive virtual environment, human-stimuli are not static, imaginary, or implied others, as is often the case in experimental psychology, but interactive and communicative others that can be manipulated in terms of outward appearance and inner disposition.

Consider how immersive virtual environments have been used to study the social effects of height. Correlational research had shown that tall people have more self-esteem and social-esteem, leading to greater career success (Judge and Cable, 2004). But, if tall people behave differently, how much of this is due to self-perception and how much is due to the expectations that others have of them? Yee and Bailenson (2007) used an immersive virtual environment to manipulate participants' self-perception, giving them either tall or short avatars. After getting accustomed to their virtual body in a virtual mirror, participants engaged a confederate (who always saw the same body) in an ultimatum game. The findings showed that participants who perceived themselves to be taller were indeed more confident. Yee and Bailenson (2007) termed this tendency for participants to conform to the behavior expected from their avatar the “Proteus Effect.” Identification with one's avatar can also lead to stereotype reduction and perspective taking. For instance, Yee and Bailenson (2006) showed that research participants who were placed in avatars of older people became less likely to stereotype elderly people. While the preceding studies manipulated participants' self-perception, the paradigm can be used for more profound interventions in participants' sensory processing. For example, using a virtual environment to simulate the embodied experience of color-blindness led to participants having increased empathy for people who were color-blind (Ahn et al., 2013).

Immersive virtual environments and other avatar methods enable researchers to manipulate the various aspects of self-perceived and other-perceived identity as well as first-person sensory experience, and do so in a manner that offers tight experimental control and high replicability (Blascovich et al., 2002). Placing research participants in virtual social scenarios abstracted from real-world situations so as to validate psychological phenomena bypasses the complications of having to create these real-world situations in the laboratory or in the field (e.g., the bystander effect; Kozlov and Johansen, 2010). So whereas the use of machine-bodied hybrids in psychological research is limited by technological bottlenecks, social interaction constraints, and the high costs associated with creating a physical android or robot (Ziemke and Lindblom, 2006), the avatar paradigm offers the opportunity to manipulate various dimensions of the body and surrounding social context in a far less constrained manner, thus offering more room for exploratory research and hypothesis testing. However, while avatar methods stress the importance of the animate body, they fall short in offering “actual” presence of a fleshy body that speaks. No amount of technological sophistication can remove participants' awareness that a virtual interaction is indeed *virtual* (Washburn, 2003). It is well established that one of the most powerful mediators of human behavior is the proximity of an actual human body, such as an authority source or victim (Milgram, 1974); digital mediation reduces proximity (Dubrovsky et al., 1991). One need only glace at people's behavior in online gaming communities to know that there are things people will do *to* and *through* an avatar that they would never do *to* and *through* an actual human body. Indeed, this fantasy element is precisely what makes virtual environments appealing (Suler, 2004).

### Intelligent virtual agents

The virtual analog to the autonomous robot or android is the intelligent virtual agent (or embodied conversational agent), an entity that involves an artificial speech and cognition source paired to a virtual body (Cassell, 2000). These agents are commercially ubiquitous (e.g., online virtual support agents; Etemad-Sajadi and Ghachem, 2015), but are also used extensively in basic and applied social psychological research (e.g., Brave et al., 2005; Gratch et al., 2007). For instance, intelligent virtual agents have been used increasingly in the domain of healthcare training, serving as virtual coaches for trauma victims (Tielman et al., 2015), mock interviewees for medical students (Carnell et al., 2015), and meditation coaches (Shamekhi and Bickmore, 2015). The intelligent virtual agent literature consistently underscores the importance of the body in human-agent intersubjectivity (with the uncanny valley phenomenon being key). For instance, agents that physically gesture in a manner associated with an underlying psychological state suggested by their speech stand a better chance at being positively evaluated by users (see Guadagno et al., 2011). More successful agents are those with the capacity to infer a user's emotional responses and physically mirror these psychological states; appropriately doing so in the context of a specific social situation allows the user to “suspend their disbelief” and engage the agent in a more fully human manner (de Melo and Gratch, 2015).

Similar to the avatar paradigm, intelligent virtual agent methodology affords the researcher the ability to create replicable, highly controlled human stimuli that can be deployed in cases where real-world stimuli are not conducive or available for experimentation. For example, virtual agents representative of various abnormal psychological conditions can provide medical students with a means of scaffolding their diagnostic acumen prior to their encountering actual patients (Kenny et al., 2009). Like with autonomous androids and robots, however, social interactions under this paradigm are fundamentally constrained by the limitations of artificial speech systems (even the most advanced conversational agents cannot fluidly adapt in human-level conversation). Contemporary intelligent virtual agents are therefore domain-specific and narrow in their capacity to socialize beyond strict conversational parameters. Moreover, as with avatars, the non-fleshiness of virtual agents presents limitations in terms of generalizing research findings to real-world situations involving actual human beings.

### Cyranoids

**Figure 1:**
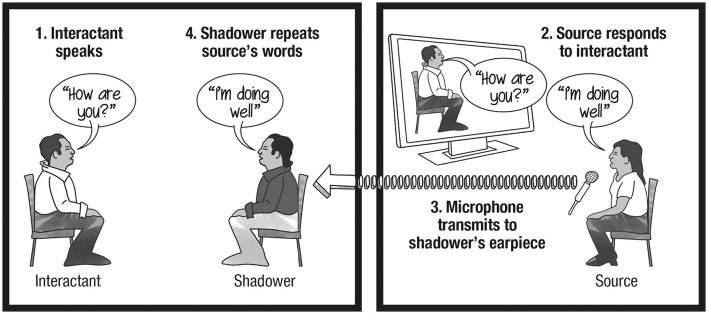
**Combining a speech source and a speech shadower to produce a cyranoid**.

Having one human speak in real-time through the body of another human is known as the “cyranoid method” (see Figure 1). The social psychologist Milgram (1992), who developed this method, trained people to **speech shadow** (i.e., replicate the words of a remote source via a covert audio-relay apparatus), thus enabling them to them ventriloquize other people's speech while engaging in face-to-face interactions with third parties. Inspired by *Cyrano de Bergerac*, Milgram referred to the resulting hybrids as “**cyranoids**.” When naïve to their interlocutor's hybrid nature, third parties reliably succumbed to what Milgram called the “**cyranic illusion**,” namely, the failure to perceive that the words of a dialog partner are not self-authored.

KEY CONCEPT 3 **Speech shadowing**

Speech shadowing entails a “speech shadower” repeating verbatim words produced by a “speech source” (usually another person) as instantaneously as possible. With practice shadowers can track the speech source with a latency of about 250 ms, about the duration of a syllable (Marslen-Wilson, 1973). Speech shadowing imitates source words more accurately than people reading transcripts of the shadowed words (Shockley et al., 2004).

KEY CONCEPT 4 **Cyranoid**

A cyranoid, first proposed by Milgram (1992), is a human body that covertly receives and repeats in real-time (speech shadow) words originating from another human. Cyranoids are hybrids, combining the body of one person with the speech of another. People who interact with a cyranoid, known as “interactants,” tend not to notice that the body that speaks does not author the words being spoken (see cyranic illusion).

The cyranic illusion's robustness is due in large part to the nature of speech shadowing. A proficient shadower is

KEY CONCEPT 5 **Cyranic illusion**

The cyranic illusion, identified by Milgram (1992), refers to the finding that people interacting with cyranoids tend not to notice that the body they are ostensibly speaking to is not authoring the words being spoken. The cyranic illusion has been shown to hold for large discrepancies between the cognition source and the speaking body, such as a child speaking the words of an adult.

able to repeat unrehearsed individual words as they are being spoken with only a few hundred ms of delay (Marslen-Wilson, 1973; Bailly, 2003), creating the appearance that one is speaking spontaneous self-authored words. Once this level of skill is achieved, shadowing requires minimal cognitive effort and the shadower can divert their attention to mimicking body language appropriate to the communicative scenario. We have orchestrated several hundred cyranoid interactions, and our expert shadowers report that cyranic conversations can be carried out with ease for long durations as they don't have to think about what to say.

Milgram died before he could carry out the research he envisioned and failed to produce a journal article detailing his use of cyranoids. The method lay dormant until we carried out the first systematic experiments on the robustness of the method (Corti and Gillespie, 2015b). Our first study compared cyranoid interactions with non-cyranoid interactions. Forty participants engaged in unscripted, face-to-face, dyadic discussions with an adult confederate who was either communicating autonomously (control condition) or shadowing the words of a remote adult source (cyranoid condition). Analysis of videos of these 10-minute interactions, debrief interviews, and post-interaction questionnaires demonstrated that the conditions were comparable from the point-of-view of the naïve participants (i.e., those in the cyranoid condition failed to detect that their interlocutor was a cyranoid), thus providing evidence for the cyranic illusion.

Our second study examined whether the cyranic illusion would hold if there were a stark incongruence between the speech source and the shadowing body. Seventy-two participants naïve to the cyranoid method were randomly assigned to small groups tasked with interviewing a confederate face-to-face for 20 min. The confederate was either: an autonomous 12-year-old boy, an autonomous 37-year-old university professor, a cyranoid composed of the boy speech shadowing for the professor, or a cyranoid composed of the professor speech shadowing for the boy. Again, video analysis of the interactions, debrief interviews, and post-interaction questionnaires showed that the cyranic illusion still held; participants believed they were interacting with an autonomous person despite incongruities between the body they saw and the words they heard. Futhermore, we were able to show how participants interacted differently with the same speech source (child or professor) depending on the body that spoke (Corti and Gillespie, 2015b).

### Echoborgs

Taking the cyranoid methodology one step further, we combined speech shadowers with chat bots in order to create “**echoborgs**”: hybrid entities consisting of a human body paired to an artificial speech source (Corti and Gillespie, 2015a; for a video demonstration of the method, see Corti and Gillespie, 2015c). Our echoborg research was born out of a motivation to push the limits of the cyranic illusion. We found that echoborgs could “pass” as autonomous humans when conversing face-to-face with naïve interactants for 10 min. The caveat is that participants tended to see the echoborgs as either having a personality disorder (e.g., social anxiety) or as acting in some way (albeit spontaneously authoring their own words). These peculiarities cannot be attributed to the medium (a human body that speaks), but rather are due to the idiosyncrasies of the speech source (our studies primarily made use of the chat bot *Cleverbot*; Carpenter, 2015).

KEY CONCEPT 6 **Echoborg**

An echoborg is a human that covertly receives and repeats in real-time (speech shadow) words originating from an artificial speech source. Corti and Gillespie (2015a), the developers of the echoborg method, demonstrated that people interacting with an echoborg fail to detect that the speech source is artificial (see cyranic illusion). Echoborgs can be used to assess how people interact with artificial speech sources when they firmly believe that they are engaged in a human-human interaction.

The echoborg methodology is an inversion of the tele-operated and Wizard of Oz techniques. Whereas the tele-operated paradigm within android science has been used largely as a means of circumventing the bottlenecks in artificial intelligence (Nishio et al., 2007b), the echoborg paradigm makes the reverse trade-off: privileging absolute bodily realism over human-level speech and cognitive sophistication. Likewise, as the Wizard of Oz technique is intended to prime research participants to believe that they are interacting with a fully autonomous agent when in fact the agent is being operated by a human, the echoborg method generates the illusion that one is interacting with an autonomous person as opposed to an agent in control of the words spoken by a human body. Though we have yet to attempt creating such a hybrid, we imagine future echoborg research might include the agent giving behavioral cues to the speech shadower, thus extending the agent's influence within an echoborg beyond mere word authorship.

We argue that the echoborg method is currently the only means of examining human-agent interaction under the full social psychological conditions of human-human interaction, an affordance which grants researchers the ability to investigate how a fleshy human body fundamentally alters the experience of interacting with artificial intelligence and to what extent a real human body is *necessary* in order to elicit certain patterns of human communication. To support this claim, we investigated the phenomenon of conversational repair using echoborgs and less anthropomorphic mediums (Corti and Gillespie, 2016; for an overview of conversational repair, see Schegloff, 1992). We found that people more frequently initiate verbal repairs of misunderstandings when they encounter a human-bodied agent-interlocutor (vs. a screen-based agent-interlocutor) as well as when they *believe* they are speaking to an autonomous human (vs. an agent), and most frequently initiate repairs when both of these conditions are met. In other words, people exerted the most “intersubjective effort” toward establishing conversational common ground when they interacted with a human body that they believed was an autonomous human (when, in fact, the speech source was always an agent).

Echoborg methodology can thus be operationalized to test human-agent intersubjectivity against human-human benchmarks. Human-agent interaction researchers have argued that “the goal of human-agent interaction […] should not be a believable agent; it should be a believable interaction between a human and agent in a given context” (Cassell and Tartaro, 2007, p. 407). In other words, what really *counts* in human-agent interaction, insofar as the agent has been designed to mimic the conversational sociality of a human, is not so much the stand-alone cognitive sophistication or aesthetic quality of the agent, but rather its ability to scaffold a quality of intersubjectivity with its human user that is indistinguishable from that seen in mundane human-human interaction. Since, as we have shown (Corti and Gillespie, 2016), the fleshy human body (and the associated belief that the mind is human) elicits complex intersubjective behaviors from human interactants to a greater degree than that elicited by non-human mediums, the echoborg method can be used to test how various agents perform when human-level intersubjectivity is expected from users. The more successful conversational agents will be those that can achieve and maintain a fluid, non-domain-specific conversation with a human interactant in a context wherein the interactant, by virtue of their *believing* they are interacting with another human mind, *assumes* human-level intersubjectivity. The echoborg method can create such contexts by-way-of placing a real human body in front of the human interactant.

### Methodological and conceptual caution with cyranoids and echoborgs

While the possibilities for recombining bodies and speech sources to form cyranoids and echoborgs are exciting, methodological and conceptual caution is required. There is always an element of latency with speech shadowing, and this is most evident with echoborgs, whose speech latency is largely a function of both the means by which an interlocutor's words are imputed into the chat bot and the apparatus that relays the chat bot's words to the shadower (see Corti and Gillespie, 2015a). Such delays can have unstable communicative significance. For example, a delay preceding a thoughtful utterance has a different social significance than a delay in participating in joint laughter. Though speech shadowers reflexively imitate the emotive and gestural elements (e.g., intonation, tone, emphasis) of their source's vocal delivery to a certain degree (a phenomenon known as “phonetic convergence”; Pardo et al., 2013), a shadower's ability to replicate the full suite of vocal gestures generated by their source is naturally limited. Thus, while the semantic and syntactic content is fully provided by the speech source, some of the speech style and all of the source's nonverbal behaviors (e.g., eye gaze, facial expression, gestures, posture) are provided by the shadower (unless, as stated above, the source provides the shadower with certain behavioral cues). Without appropriate controls and a well-practiced shadower, the nonverbal communication of a cyranoid or echoborg can be at odds with the words provided by the source, creating an unintentionally inauthentic persona. These practical challenges can also make interpreting experimental results difficult. It is important to remember that cyranoids and echoborgs are *hybrids* and should not be thought of as a singular nervous system (or artificial agent) in complete control of a body. Rather, cyranoids and echoborgs are two or more separate nervous systems (or agents) combining to control different components of a seemingly singular social persona.

## The human body in communication: a conceptual framework

While studies examining tele-operated robots and androids, fully autonomous robots and androids, avatars, and intelligent virtual agents consistently reveal the significance of the visible body, it is only with the cyranoid and echoborg methods that fleshy human bodies are used as mediums for various speech sources. Thus, it is only with these two methods that we can parse the intersubjective processes uniquely elicited by conversing with a *real* human body.

Descartes' (1641/1985) over-sharp separation between the body and cognition has been repeatedly undermined by empirical research showing that cognition is, in many subtle ways, inextricably bound to the body (Damasio, 2006) and evolutionary processes (McKeown, 2013). But, Descartes' second error, to neglect the importance of social interaction for real-time dynamics of cognition (Gillespie, 2006), remains widespread (Farr, 1996). Even in the embodied cognition research paradigm (which champions the importance of the body), the body *for* the other is usually overlooked (Schilbach et al., 2013). Although the body is undoubtedly the fleshy underpinning of cognition, it is also the visible marker of a cognition source to other people; it is the medium through which people engage with that cognition source and in doing so attribute consciousness to that cognition source (Graziano, 2013). In this sense, the body shapes the perception of the mind because it is the gateway to the mind (Biocca, 1997). The cyranoid and echoborg methodologies are not useful for understanding the direct links between the body and cognition, however they are uniquely powerful for understanding how the body, as a medium of communication, mediates cognition.

Face-to-face interaction between two people needs to be conceptualized on at least three levels identified by Ichheiser (1949a, p. 59) in his “framework of images in human relations.” At the first level is what each person thinks about themselves. At the second level is what each person thinks about the other person. Finally, at the third level is what each party assumes that the other thinks about them. Although this framework can be extended to higher levels of complexity, additional levels are difficult to address methodologically (Gillespie and Cornish, 2010). Table 2 maps this three-level framework onto research questions operationalized using the cyranoid and echoborg methods.

**Table 2:** **A conceptual framework for studying the body that speaks**.

**Level**	**Conceptualization**	**Research questions**
1	Self's perspective on self	How does the source's perception of their new body mediate their cognition, action and understanding of other people with similar bodies?
2	Other's perspective on self	How does the interactant's perception of the body that speaks mediate their understanding of what is said?
3	Self's perspective on other's perspective on self	How does the source pick up on, conform to or react against the interactant's expectations about the body that speaks?

### Level 1: self-perception and identification

The first level concerns how the body contributes to self-perception. Longstanding theory (e.g., Mead, 1913) and research (e.g., Bem, 1972) suggests that a key component of selfhood is self-perception. That is to say, we don't only know ourselves from the inside, we also observe ourselves from the outside in the same way that we observe others. This raises the question: if people observe themselves in a different body, might they start to think and behave differently?

This question has been examined using avatars in virtual environments. As reviewed above, this research has found that people do indeed seem to behave in a way that conforms to the body of their avatar (Yee and Bailenson, 2007) and that being in the virtual body of another can facilitate identification with people who routinely live in a given body type (Yee and Bailenson, 2006). Using the cyranoid methodology, research could try to replicate these findings in fleshy face-to-face interactions. Although it would be technically challenging to blind the shadower to the condition (avatars are blind by default), the upshot would be convergent validity with even higher mundane realism.

### Level 2: the cyranic illusion, impression formation, and stereotyping

The second level focuses on the meaning of the body for interactants, and specifically how it mediates their cognition and behavior. Four main lines of research are evident at this level.

First, there is the phenomenon of the cyranic illusion. Our research has shown that the illusion extends to a child body speaking the words of a professor and to an adult body speaking the words of a chat bot. This illusion, however, can be pushed in other directions. Milgram (1992) himself wondered whether a super-cyranoid could be created, with several sources (e.g., multiple human experts) providing suggestions to the shadower who would be selective in using the assistance. Again, we could ask: would interactants recognize the full nature of their interlocutor's speech source? Assistive technologies for cognition are developing rapidly such that it might not be too long before people routinely have access to in-ear cognitive support (O'Neill and Gillespie, 2015). The cyranoid and echoborg methods could enable research to get ahead of technological development to examine how augmented humans would function and be perceived in social settings. Another direction to push the cyranic illusion would be to introduce familiarity as a variable: how long would it take someone to notice either that an unfamiliar body was shadowing their romantic partner, or, that their romantic partner was shadowing an unfamiliar cognition source? We could thus use the cyranoid method to parse the role of the body and “mind” in close relationships.

Second, the cyranoid and echoborg methods can be used to study the communicative significance of nonverbal gestures in unscripted face-to-face conversation. Research on nonverbal gestures has had to rely on naturalistic studies, scripted interactions (e.g., Burgoon et al., 1984) or virtual agents (Georgescu et al., 2014). If participants blind to the condition or advanced chat bots were speech sources for human speech shadowers who engaged, according to condition, in the given nonverbal behavior (e.g., eye contact, posture, or facial expression), then any variability between the conditions in either the behavior of interactants or the impressions they reported would be attributable to the gesture manipulation.

Third, the cyranoid and echoborg methods can be used to study stereotyping based on body cues in unscripted face-to-face conversations, providing powerful convergent validity to the numerous studies that have activated stereotypes with priming (e.g., Wheeler and Petty, 2001). Either participants blind to the condition or advanced chat bots could be speech sources for bodies differentiated by weight, height, skin color, attractiveness, accent, gender, visible disability, or bodily markings (e.g., piercings, tattoos and birthmarks). Again, the differences perceived by interactants would be attributable to the body that speaks, thus providing robust evidence for the impact of stereotypes in unscripted face-to-face interaction.

Finally, humans do not interact with artificial intelligence in the same way that they interact with other humans. However, it is difficult to identify the source of this difference. Do these differences arise from limitations in the artificial intelligence systems themselves? The Wizard of Oz paradigm can be used to overcome the limitations of artificial intelligence, essentially endowing mechanical or virtual bodies with human intelligence. But, still the pattern of interaction is different. Is this because the mechanical or virtual body triggers certain stereotypes among human interactants? The most robust methodology for examining how humans would interact with artificial intelligence under the assumption that it is a human, we argue, is the echoborg technique.

### Level 3: orienting to the perspective of the other and behavioral confirmation

According to Ichheiser (1949b) one of the main biases in social interaction is that people fail to see how they themselves contribute to their own social environment; we don't encounter people “as they are,” rather, we meet them “as they respond to us.” The unique social world that rises up to meet each of us is furnished with other people's expectations that are a function of our bodies, dress, gestures, words, actions, reputation, and social history. Moreover, Ichheiser (1949b, p. 29) observed that we have a “tendency” to adjust our behavior to respond to, and even conform to, these expectations that other people have of us; thus further contributing to the peculiar social worlds that each of us inhabit.

Since Ichheiser's insightful suggestions, research has shown that we do indeed have a tendency to conform to the expectations others have of us, even at an unconscious level (Chen and Bargh, 1997). In a classic study Snyder et al. (1977) randomly assigned men and women, who were strangers to each other, to have a telephone conversation. The men were presented with a Polaroid photograph of either an attractive or unattractive woman who they were led to believe was their conversation partner. Blind rating of the men's utterances showed that the men who thought they were conversing with attractive women were more sociable, sexually warm, interesting, independent, sexually permissive, bold, outgoing, humorous, and socially adept. Moreover, blind rating of the women's utterances (all the women were unaware of the photographs) showed that the women whose male partners had been led to believe they were attractive spoke with more confidence, greater animation, greater enjoyment and greater fondness of their conversation partner. Since this classic study, however, it has been impossible to get closer to face-to-face communication than a telephone call or some other distal form of mediated interaction. Using the cyranoid methodology, with speech sources blind to the peculiarity of the body that speaks their words, the effect of behavioral confirmation could be studied in unscripted face-to-face conversations.

What about the expectations that interaction partners might have toward a mechanical body? People interacting with artificial intelligence do less mentalizing (Chaminade et al., 2012) and initiate fewer verbal repairs of misunderstanding (Corti and Gillespie, 2016). Would a human speech source, blind to the mechanical body that they were speaking through, be able to detect these lowered expectations? Instead of behavioral confirmation (i.e., the speech source behaving more like a chat bot), might there be resistance to these lowered expectations (Jussim, 1990)? Maybe the speech source would attempt, at an unconscious level, to demonstrate their humanity and complexity. Or, perhaps humanity has to be seen in the body (and thus be expected) in order to be given the space to flourish. These unexplored research questions are made tractable by the echoborg method.

## Conclusion: the body that speaks

Information does not flow freely around the globe; each micro-step entails reconstruction mediated by communication processes (Habermas, 1981). Often these communication processes relate to the peculiar dynamics of websites, television programs, newspapers, books, and so on. But even in our mass mediated world, the most important flows of information occur face-to-face; family get-togethers, teaching, team meetings, job interviews, salary negotiations, business transactions, corporate mergers and international relations are all underpinned by face-to-face meetings. Accordingly, despite the proliferation of communication technologies (e.g., email, telephone, video conferencing, avatars), human bodies are moved around the globe at an unprecedented rate (Urry, 2012).

It is well established that individual cognition is grounded in the body (Barsalou, 2008), but thinking in everyday life often occurs between people in conversation (Schilbach et al., 2013; Marková, 2016). At this more social point in the knowledge construction process the body re-enters as particularly important, not because it is the fleshy underpinning of cognition, but because it is the medium that speaks. Our bodies are important because they shape our self-perception, guide our perception and expectations of others, and form the basis for the expectations that others have of us and to which we orient and respond. Some bodies have authority and voice, while other bodies are ignored and not listened to—processes which feed-forward into self-perceptions and reinforced expectations, thereby potentially distorting knowledge construction (Habermas, 1970).

“The medium is the message,” McLuhan (1964, p. 9) wrote, “because it is the medium that shapes and controls the scale and form of human association and action.” The body, as our primary medium, will always give precedence to face-to-face communication: the meeting of bodies, our primordial interaction, is the wellspring of social emotions and trust (Marková and Gillespie, 2008). It is in this fleshy interface that social meanings are made (Farr, 1997). Within this meaning-making process the body is central, not as a speech source, but, as a social cue. The speech source needs to combine with a body to speak, and the impression made on the interlocutor is a function both. Accordingly, it is not only the speech source that communicates, but also the body that speaks.

## Author contributions

AG produced the first draft and KC contributed substantial comments and text.

### Conflict of interest statement

The authors declare that the research was conducted in the absence of any commercial or financial relationships that could be construed as a potential conflict of interest.
